# Neutrophil NETworking in ENL: Potential as a Putative Biomarker: Future Insights

**DOI:** 10.3389/fmed.2021.697804

**Published:** 2021-07-14

**Authors:** Smrity Sahu, Keshav Sharma, Maryada Sharma, Tarun Narang, Sunil Dogra, Ranjana Walker Minz, Seema Chhabra

**Affiliations:** ^1^Department of Immunopathology, Postgraduate Institute of Medical Education and Research, Chandigarh, India; ^2^Department of Otolaryngology and Head and Neck Surgery, Postgraduate Institute of Medical Education and Research, Chandigarh, India; ^3^Department of Dermatology, Venereology and Leprology, Postgraduate Institute of Medical Education and Research, Chandigarh, India

**Keywords:** ENL, neutrophils, biomarker, NETs, biology, reprogramming, exosomes

## Abstract

Erythema nodosum leprosum (ENL), also known as type 2 reaction (T2R) is an immune complex mediated (type III hypersensitivity) reactional state encountered in patients with borderline lepromatous and lepromatous leprosy (BL and LL) either before, during, or after the institution of anti-leprosy treatment (ALT). The consequences of ENL may be serious, leading to permanent nerve damage and deformities, constituting a major cause of leprosy-related morbidity. The incidence of ENL is increasing with the increasing number of multibacillary cases. Although the diagnosis of ENL is not difficult to make for physicians involved in the care of leprosy patients, its management continues to be a most challenging aspect of the leprosy eradication program: the chronic and recurrent painful skin lesions, neuritis, and organ involvement necessitates prolonged treatment with prednisolone, thalidomide, and anti-inflammatory and immunosuppressive drugs, which further adds to the existing morbidity. In addition, the use of immunosuppressants like methotrexate, azathioprine, cyclosporine, or biologics carries a risk of reactivation of persisters (*Mycobacterium leprae*), apart from their own end-organ toxicities. Most ENL therapeutic guidelines are primarily designed for acute episodes and there is scarcity of literature on management of patients with chronic and recurrent ENL. It is difficult to predict which patients will develop chronic or recurrent ENL and plan the treatment accordingly. We need simple point-of-care or ELISA-based tests from blood or skin biopsy samples, which can help us in identifying patients who are likely to require prolonged treatment and also inform us about the prognosis of reactions so that appropriate therapy may be started and continued for better ENL control in such patients. There is a significant unmet need for research for better understanding the immunopathogenesis of, and biomarkers for, ENL to improve clinical stratification and therapeutics. In this review we will discuss the potential of neutrophils (polymorphonuclear granulocytes) as putative diagnostic and prognostic biomarkers by virtue of their universal abundance in human blood, functional versatility, phenotypic heterogeneity, metabolic plasticity, differential hierarchical cytoplasmic granule mobilization, and their ability to form NETs (neutrophil extracellular traps). We will touch upon the various aspects of neutrophil biology relevant to ENL pathophysiology in a step-wise manner. We also hypothesize about an element of metabolic reprogramming of neutrophils by *M. leprae* that could be investigated and exploited for biomarker discovery. In the end, a potential role for neutrophil derived exosomes as a novel biomarker for ENL will also be explored.

## Introduction

Leprosy is a chronic granulomatous infectious disease caused by an intracellular pathogen *Mycobacterium leprae* where the clinical spectrum of the illness is dictated by the host immune response to the pathogen (1). The clinical course of leprosy is punctuated by dynamic, unpredictable, painful immune-mediated inflammatory episodes known as reactions. These leprosy reactions are a major clinical concern and up to 50% of leprosy patients experience at least one reaction during their lifetime (2). Erythema nodosum leprosum (ENL)/type 2 reaction (T2R), an acute nerve-destructive immune exacerbation, is encountered in patients with borderline lepromatous (BL) and lepromatous leprosy (LL) and can occur before, during, or after multi-drug treatment (3). It is estimated that annually over 50,000 newly diagnosed leprosy patients are at risk of developing ENL (4). These patients classically present with tender, erythematous, evanescent subcutaneous nodules along with multisystemic illness manifesting as neutrophilic leukocytosis, fever, sepsis-like malaise, iritis, iridocyclitis or conjunctivitis, arthritis or arthralgia, bone pain and tenderness especially tibial tenderness, dactylitis, lymphadenitis, oedema of extremities, orchitis, and neuritis ultimately leading to significant sequelae associated with peripheral nerve dysfunction. The clinical manifestations of ENL can occur in any of 3 patterns viz. single acute episodes, recurrent episodes, and chronic disease with varying grades of severity (5). ENLIST ENL Severity Scale (EESS) a 10-point severity scale system is a validated tool used in clinics to assess ENL severity (6). Atypical clinical manifestations like pustular, bullous, necrotic, and annular ENL have also been reported and sometimes T2Rs may present with rheumatologic complaints leading to misdiagnosis. Besides a high index of clinical suspicion, point-of-care diagnostic assays or biomarkers are needed that could be used by leprosy workers in the field to confirm the diagnosis and initiate treatment to avoid deformities and disabilities.

The incidence of this multi-system inflammatory disorder is certainly on the rise with the increasing number of multibacillary cases (7). The bactericidal effect of Multi-drug therapy (MDT) or other antibiotics on *M. leprae* results in the release of an enormous amount of bacterial breakdown products that are recognized by the innate immune system as antigens resulting in activation of pro-inflammatory cytokines (7, 8).

These reactions are often diagnosed late in the absence of reliable, early diagnostic tests. Attempts to identify general biomarker signatures (Table 1) for ENL are often hampered due to patient heterogeneity, different male/female ratios across various studies, lack of sufficient power of many studies due to low number of disease subjects, long incubation times of leprosy and lack of appropriate control groups (LL/BL patients without ENL, instead of healthy controls) (9). There is an unmet need for predictive correlates as a diagnostic tool allowing early diagnosis of ENL during MDT in high-risk leprosy patients that is simultaneously useful for discriminating patients with ENL from those with Type-1 reactions.

**Table 1:** List of putative biomarkers for Erythema nodosum leprosum/Type 2 reaction in leprosy patients.

**S No**	**Biomarkers**	**Total No. of subjects**	**Sample type**	**Applied technique**	**Remark**	**Place of study**	**Year (References)**
01	IL-7, PDGF-BB, IL-6, VEGF	ENL-10Non-reactional LL-10	Plasma	Luminex	Elevated plasma IL-7 and PDGF-BB levels can act as a putative biomarker for ENL when compared with Type 1 reaction leprosy patients	Goiania city, Brazil	2009 (10)
02	Alpha 1-Acid glycoprotein (AGP)	ENL (Untreated)-6ENL (Treated)−6Non-reactional LL-6RR-7BT-5BB-4BL-5HC-9	Serum	ELISA,2D electrophoresis MALDI-TOF	Increased serum levels of AGP in untreated ENL patients can be used as biomarker to differentiate these patients from non-reaction patients	Madurai, India	2010 (11)
03	Alpha 1-Acid glycoprotein (AGP)	ENL-32RR-17Non-reactional LL-39	Serum Skin biopsies	ELISAqPCR	The higher AGP levels as reflected in the serum profile, in active reaction patients than in controls with no reaction could be an early indication of disease progression.	Delhi, India	2015 (12)
04	CD 64 on neutrophils	ENL (Untreated)−11ENL (Treated)-10LL-8HC-10	Whole blood and skin biopsies	Flowcytometry (Whole blood)qPCR (Skin Biopsy)	CD64 was significantly down regulated in ENL lesions after beginning thalidomide treatment. Increased CD64 expression on the surface of neutrophils in circulation as well as in biopsy can be used as biomarker for ENL.	Rio de Janeiro, Brazil	2016 (13)
05	Pentraxin-3 (PTX-3)	ENL (MB)-27RR (MB)-11RR (PB)-9MB-19PB-15	Serum	ELISA	PTX-3 levels were assessed as a biomarker for active ENL which was suppressed after thalidomide treatment	Rio de Janeiro, Brazil	2017 (14)
06	LID-1	ENL-41RR-119Reaction free-292	Serum	ELISA	Higher serum levels of LID-1 in RR patients isolates them from the non-reaction patients	Goniana city, Brazil	2017 (15)
07	Activated B cells and tissue-like memory B (TLM-B) cells	ENL-41Non-reactional LL- 30	Whole blood	Flowcytometry	Increase in activated B cells and reduction in tissue-like memory B cells in ENL as compared to LL patients can be used as biomarker for ENL.	Addis Ababa, Ethiopia	2017 (16)
08	C1q (C1qA, C1qB, and C1qC)	ENL (untreated)- 30Non-reactional ENL-30	Whole blood and skin biopsies	qPCR, ELISA	Circulating C1q in the peripheral blood of untreated ENL patients was significantly lower compared to LL patient controls. C1qA and C1qC gene expression were substantially increased in the skin biopsies of untreated ENL patients compared to LL controls.	Ethiopia (Addis Ababa)	2018 (17)
09	Neutrophils in dermis	ENL-10MB-23	Skin biopsies	Histopathology	Elevated number of neutrophils can be used as a biomarker for the ENL.	Denpasar, Bali	2018 (18)
10	IgM, IgG1, C3d	ENL-29RR-35MB-51HC-15	Serum	ELISA	Association of increased IgM, IgG1, and C3d-associated immune complexes could be used to estimate risk of developing reactions.	Natal, Brazil	2019 (19)
11	Low density neutrophils (CD14– CD15+)	NA	Whole blood	Flowcytometry	Increased circulating activated low density neutrophils are associated with ENL.	NA	2020 (20)
12	IL10 R1 on neutrophils	ENL-28Non-reactional BL/LL-28	Whole blood	Flowcytometry	IL10R1 was used as a potential indicator of ENL. The levels of IL10R1 were detected in measurable amount in ENL patients as compared to BL/LL patients or healthy donor.	Rio de Janeiro, Brazil	2021 (21)
13	Neutrophil-lymphocyte ratio	ENL-56Reversal reaction- 42TL-2556-BL42- LL	Whole blood	Automated blood counter	Neutrophil-lymphocyte ratio could be a potential biomarker for diagnosis of leprosy reaction and useful for discriminating patients with type 2 reactions from those with type 1 leprosy reactions.	Rondonia, Brazil	2020 (22)
14	IL-6	ENL-15MB without ENL-16HC-15	Serum	Cytokine bead array (Flowcytometry)	High-serum levels of interleukin-6 were observed during ENL, primarily in patients with more severe disease and levels decreased after specific therapy. Elevated levels of IL-6 observed during ENL episodes can be used as a biomarker.	Rio de Janeiro, Brazil	2021 (23)

*IL, Interleukin; PDGF-BB, Platelet-derived growth factor BB; VEGF, Vascular endothelial growth factor; ENL, Erythema nodosum leprosum; LL, Lepromatous leprosy; RR, Reversal reaction; BT, borderline tuberculoid; BB, borderline borderline; BL, borderline lepromatous; HC, Healthy control; ELISA, Enzyme linked immune-sorbent Assay; 2D-2, Dimensional; MALDI-TOF, Matrix-assisted laser desorption/ionization-time of flight; qPCR, Quantitative polymerase chain reaction; LID-1, Leprosy IDRI diagnostic 1; MB, Multibacillary leprosy; Ig, Immunoglobulin; EC, Endemic control; TLM-B, Tissue-like memory B cells*.

Neutrophils, ascribed as warriors against pathogens and critical actors in the innate immune system with high pro-inflammatory activity constitute the dominant immune cell population in human blood with ~10^11^ neutrophils generated every day in the bone marrow (24, 25). They are the first responders to infection and inflammation, providing a key pivot between resolution or propagation of collateral damage that can result in multi-system organ failure. They possess an enormous armamentarium to counter pathogens. These cells are endowed with huge microbicidal potential by virtue of their phagocytic capacity, ability to produce reactive oxygen intermediates, a rich array of granule-derived lytic enzymes and a tendency to release neutrophil extracellular traps (NETs). A vast body of literature shows that neutrophil dysfunction can contribute significantly to sepsis and a variety of autoimmune diseases (24, 26, 27). Neutrophils can promote cancer progression by facilitating matrix remodeling, stimulating angiogenesis, and by disabling T-cell-dependent antitumor immunity (28).

ENL is considered a neutrophil-mediated immune-complex reactional state, histologically defined by leukocytoclastic vasculitis and prominent neutrophilic infiltrate throughout the dermis and subcutis during the acute stage that is gradually replaced by lymphocytes with the evolution of the lesion (3–6). Neutrophils are considered as the “signature cell” in T2R that contribute to ENL-associated multi-systemic inflammation in multiple ways (29). Neutrophils circulating in peripheral blood of multibacillary leprosy patients are heavily bacillated, with no apparent sign of systemic inflammation (30). These neutrophils are effectively cleared of bacilli only after some months of multidrug therapy (31). A variety of neutrophilic functional abnormalities including enhanced spontaneous defective random migration, chemotaxis and chemokinesis, and increased apoptosis associated with increased production of pro-inflammatory cytokines (32) has already been demonstrated in ENL patients suggesting the role of neutrophils as active effector players in T2R (32–36).

In the present review, novel aspects of neutrophil biology and their links to ENL pathophysiology are discussed, highlighting their clinical usefulness as a potential biomarker.

## Neutrophil Biology

### Neutrophil Generation and Activation: Birth and Evolution of “Forefront Warriors”

Steady-state granulopoiesis is stringently regulated by various factors including PU.1, CCAAT/enhancer binding protein α and growth factor independence 1. Normal homeostatic neutrophil counts are maintained by expression of granulocyte colony stimulating factor (G-CSF) tied to interleukin (IL)-23 and IL-17 production by lymphocytes. The short-lived CD66b^+^CD117^−^CD34^−^ neutrophil precursors mature rapidly inside the bone marrow and are released into the peripheral blood where the expression of membrane-bound receptors such as CXCR4 and CD117(c-kit) and integrin α4β1 (VLA4) gradually decreases while expression of toll-like receptor 4 (TLR4) and CXCR2 and other chemokine receptors progressively increases (37–39). Throughout granulopoiesis, chemokine receptors act in concert with selectins and β2 integrins in a short feedback circuit to regulate the circadian fluctuations of circulating neutrophils.

During acute or chronic infection, “*emergency granulopoiesis*” occurs as the earliest protective innate response, with increased production of IL-23 and IL-17 in the inflamed milieu providing increased G-CSF and IL-6 leading to appearance of immature neutrophils with lower buoyancy in circulation, better defined as Low Density Neutrophils (LDNs) (40).

Neutrophilic leukocytosis as an indicator of systemic inflammation is one of the earliest features noted in ENL patients. Among various parameters used for monitoring neutrophil counts, the neutrophil-to-lymphocyte ratio (NLR) has been recognized as a unique, stable marker reflecting underlying acute inflammatory response. NLR is considered an easy, inexpensive, and reproducible parameter associated with clinical outcome and disease severity (41–45). In recent years, its role as an independent prognostic factor for neoplasms and as an inflammatory biomarker in various acute and chronic cardiovascular/metabolic/ infectious/inflammatory diseases has been increasingly established (22, 46, 47).

Gomes et al. reported a mean NLR value of 9.7 ± 2.4 (*p* <0.001) for ENL diagnosis that easily differentiated ENL patients from those with Type 1 reaction with high validity cut-off of 2.95, high diagnostic accuracy (accuracy 78.0%, sensitivity 81.0%, specificity 74.0%) and an area under curve value of 0.796 (22), higher than those observed for other infectious diseases (48–50).

Oliveira et al. have already investigated the potential of *M. leprae* to induce neutrophil-mediated secretion of pro-inflammatory cytokines subsequent to their treatment with *M. leprae ex vivo* (32). Their observation reiterated the role of neutrophils as effector cell in T2R.

The neutrophil surface expression of CD64 (FcγRI), a marker of neutrophilic activation, is upregulated by a direct effect of interferon-gamma (IFN-γ) and G-CSF on precursor cells in bone marrow. Increased CD64 expression on circulating neutrophils as well as increased mRNA expression *in situ* in ENL skin lesions has been correlated with severity and advocated as a prognostic biomarker for ENL (13).

Bioinformatic pathway analyses of the gene expression profiles from ENL skin lesions have observed a role of an integrated axis comprising of TLR2/FcR activation/neutrophil migration/inflammation as a mechanism of neutrophil recruitment in ENL providing a deeper insight into neutrophil biology (51). Transcriptomic analysis has shown elevated expression of genes involved in neutrophilic degranulation and activation (52, 53). Global transcriptional profiling of peripheral blood mononuclear cells via microarray showed 517 differentially expressed genes revealing a granulocytic gene signature with significant involvement of the innate immune system (54).

Acute systemic inflammation gives rise to three different subsets of neutrophils in circulation viz., conventional nuclear-segmented neutrophils (CD16^bright^/CD62L^bright^), banded neutrophils (CD16^dim^/CD62L^bright^), and a third subset comprising of CD16^bright^/CD62L^dim^ neutrophils exhibiting immunosuppressive properties (38).

The presence of a unique subset of IL-10R1 expressing neutrophils in peripheral circulation and *in situ* in skin lesions of ENL patients and its clinical usefulness indicating disease outcome has also been demonstrated (21).

Apart from these inflammatory neutrophils, varied neutrophil subsets based on survival time, density, NET-releasing capacity, receptor expression profile, and functions have been observed (55).

Defined in 1986, a subset of neutrophils was found to be co-segregated with monocytes in the density gradient isolation and termed as LDNs based on their density being <1.07 gm/ml. These LDNs are comprised of a broad cell population with both pro-inflammatory and anti-inflammatory properties displaying mature hyper-segmented, as well as immature banded, nuclei. Hassani et al. have defined these LDNs as CD16^dim^/CD62L^high^ banded neutrophils exhibiting enhanced bacterial containment, spontaneous NETs generating ability, and the capacity to supress T-cell proliferation (56). LDNs tend to express increased levels of CD66b, CD11b, and CD35 in stimulated state in comparison to normal density neutrophils (57, 58). Studies in Systemic Lupus Erythematosus have suggested LDNs to be a distinct neutrophil subset with an increased level of copy number alterations, losses of heterozygosity and microsatellite instability, distinct proteomic, and biomechanical properties impacting their ability to travel through the vasculature (56, 59–62).

It is tempting to speculate that *M. leprae* has the potential to alter the immunomodulatory capacity of neutrophils resulting in the shift to lower buoyancy that could be related to maturation stage/degranulation status/activation level of neutrophils during ENL. Elucidation of the molecular underpinnings of the role played by LDNs in ENL pathophysiology could provide better insight into their use as biomarkers in clinical practice.

Thus, studying and monitoring neutrophil phenotypic, density, and functional heterogeneity for better assessment of immune response and severity of ENL pathology holds promise in their exploration as ENL biomarkers.

### Neutrophil Cytoplasmic Granules and Their Contribution Toward Phenotypic Heterogeneity and Functional Versatility: The Armamentarium

After stimulation by diverse stimuli, neutrophils mobilize different granules including primary/azurophilic, secondary/specific, and tertiary/gelatinase granules that subsequently degranulate releasing various compounds that modulate neutrophil trafficking and migration, and also facilitate their interaction with other innate and adaptive immune cells, thus playing an important role in inflicting collateral tissue damage. These granules are acquired sequentially during neutrophil maturation in bone marrow. The release of granular protein is a tightly regulated receptor-coupled process, mediated by distinct signaling events for each granule type, allowing the selective, differential, hierarchical mobilization of distinct subsets with the tertiary granules being the most readily mobilizable upon activation followed by specific granules, the primary ones bringing up the rear. This distinct readiness of different granule subsets toward degranulation modulates neutrophil heterogeneity and functional versatility by altering the neutrophil cell-surface protein composition that generates different cellular phenotypes underlying remarkable neutrophil plasticity (28, 53, 63–65).

These released granule matrix proteins act as mediators of neutrophil orchestration of innate and adaptive immunity. Teles et al. have demonstrated increased expression MMP2 and MMP9 mRNA expression in skin biopsy specimens as well as their increased serum levels in ENL patients. MMP3 has already been implicated in mediating vasculitic ENL processes (66, 67). Pentraxin-3, stored in specific granules rises quickly after degranulation and has already been shown to be associated with the exacerbation of inflammation in ENL (14).

These released contents of neutrophil granules and surface expression level of various markers of neutrophil activity i.e., CD66b (specific granule) and CD11b (tertiary granules) could be explored for their role in ENL progression.

### Neutrophil Extracellular Traps: Extra Armor

After encountering micro-organisms, neutrophils destroy them intracellularly as well as extracellularly: in their fight with microbes, dead neutrophils provide chromatin and proteins to form NETs and, live neutrophils create a cytoneme network (68).

NETs play a role in autoimmunity and thrombosis by accelerating the inflammatory response either as danger-associated molecular patterns or complement activators (69–76). They have not only been implicated as drivers of inflammation, but are also linked to resolution of inflammation, thus emerging as a double-edged sword and making them promising targets for future biomarker discovery.

Notably, NETs have been reported to be associated with disease-specific bioactive proteins loaded onto them (70). Intriguingly, emerging clinical and experimental studies indicate that neutrophils are able to release intrinsically and qualitatively different extracellular NETs decorated with disease-specific bioactive proteins dictated by the diseased inflammatory environment containing tissue factor, IL-1β, IL-17, and LL-37, suggesting systemic inflammation driven transcriptional-reprogramming in circulating neutrophils, which triggers *de novo* expression of disease-specific protein fingerprints which could accelerate the inflammatory response (77).

The potential of *M. leprae* to induce NETs formation *in vitro* and the contribution of these NETs in triggering ENL has already been investigated. da Silva et al. have clearly demonstrated NETs abundance in skin lesions and significantly increased DNA-histone/DNA-MPO complexes in the serum of ENL patients in addition to an increased tendency of peripheral blood neutrophils from ENL patients toward spontaneous NETs formation that showed marked reduction in all evaluated *in vivo* and *ex vivo* NETosis parameters in response to thalidomide treatment (78).

Investigating ENL-specific bioactive proteins loaded on NETs could offer insight into the pathogenesis of ENL and provide promise in developing disease-specific biomarkers therefore we advocate that the prognostic values for increased serum levels of NETs/NETs-related markers should be explored in future prospective large cohorts of multibacillary leprosy patients.

### Metabolic Reconfiguration of Neutrophils During *M leprae* Infection: Exploitation of Warriors by an External Agency

Over the last decade, a new concept of “immunometabolism” has emerged, which describes the changes that occur in the intracellular metabolic pathways in host immune cells during proliferation, differentiation, activation, and execution of effector function thereby maintaining body homeostasis (79–82). There is a vast body of literature indicating crosstalk between cellular metabolism and inflammatory/immune responses and how these two influences each other. This metabolic reprogramming of immune cells also has implications in the regulation of antitumor immune response as immune cells become tolerogenic and inefficient in cancer cell eradication (83). Tumor-elicited *c-kit* signaling triggers metabolic neutrophil modification leading to sustained levels of reactive oxygen species that suppress the functions of anti-tumor CD8+ T cells (84).

As recently as 2019, several investigators started studying the relationship between metabolism and the immune response at the cellular and organismal levels in infectious diseases and investigating how the pathogen-induced remodeling in the host cell metabolism influences the infectious disease pathogenesis and host physiology (85–87).

Our understanding of how host macrophage and Schwann cell metabolism can be altered by *M. leprae* and how these metabolic alterations can influence the outcome of infection has grown considerably over the past few years (88, 89). *M. leprae* chemically and metabolically impacts the cytosolic environment of the host cell, facilitating its persistence, proliferation, dissemination, and continued transmission (90–92). Histochemical, metabolomic, and transcriptional analyses have already confirmed that many biosynthetic pathways, such as those of cholesterol, phospholipids, and fatty acid biosynthesis are upregulated, underscoring the metabolic interface in the molecular pathogenesis of leprosy (93, 94). In Schwann cells, host cell energy metabolism is subverted, culminating in reduced mitochondrial action potential and reduced generation of reactive oxygen species resulting in allocation of as much carbon and reducing power as possible to lipid synthesis (95–97). Furthermore, pathways regulating tryptophan and iron metabolism have also been found to be defective in leprosy patients, favoring pathogen survival with high bacterial loads (97–99). Upregulation of omega-3 and omega-6 PUFA metabolism and the presence of higher levels of omega-6–derived-prostaglandin E2, lipoxin A4, and omega-3-derived lipid mediators have been reported in LL patients with high bacterial index (94). Lipid droplet accumulation represents a link between innate immune response and energy metabolism, making it worthwhile to study immunometabolic associations in leprosy (92).

Neutrophils exhibit a remarkable metabolic plasticity allowing them to survive in extremes of metabolite availability and contributing toward neutrophil population heterogeneity described in cancer-associated neutrophils and in density-gradient isolated low and high buoyancy neutrophils seen in auto-immune diseases (100). The metabolic reconfiguration of neutrophils effected by mediators released during chronic inflammatory states and carcinogenesis is an area of intense investigation and research (101).

However, the potential of *M. leprae* to induce alterations in biosynthetic pathways associated with neutrophil maturation, activation, and degranulation by virtue of its metabolic reprogramming ability remains unexplored. It is interesting to speculate that metabolic reconfiguration of neutrophils during *M. leprae* infection might differentially regulate various cytokines and chemokines as well as antimicrobial responses, resulting in detrimental overt inflammation that could ultimately determine the outcome of *M. leprae*-host interactions.

More research is needed to identify metabolic adaptations occurring in neutrophils and to link them to the corresponding exaggerated immune responses in ENL. Unraveling the cellular and molecular mechanisms involved in the regulation of metabolism in different neutrophil populations can lead to the discovery of biomarkers useful for investigating susceptibility to this life-threatening complication of leprosy, facilitating its early diagnosis and monitoring disease progression.

### Neutrophil Derived Exosomes

Exosomes are native nanovesicles that can regulate pathological processes including cancer cell immunity, immune regulation, inflammation, angiogenesis, fibrosis, and cell proliferation/differentiation/death. Emerging studies project exosomes as “high-resolution snapshots” that can efficiently and dynamically capture the complexity of cancer disease progression. However, for chronic inflammatory, autoimmune, infectious, metabolic, and fibrotic diseases exosome research is yet to mechanistically and technologically advance beyond “photograph negatives” that require extended processing to extract meaningful clinical information and benefits (85, 102–104). Diverse exosome cargo comprising of nucleic acids (DNA, RNA, miRNA), proteins, and microbial-derived components mediating cell-cell communication can potentially capture altered cell physiology, reflect disease progression/severity, stratify risks for therapeutic consideration (as in tumor patients), and inform decision-making between active surveillance or further clinical work-up.

Studies conducted so far have demonstrated the release of exosomes during extracellular and intracellular bacterial infections. The significance of these cargos in host-pathogen interactions is at a preliminary stage of investigation, and the role of exosomes in host defense and in determining disease outcomes remains unexplored (86, 87, 105–108).

Identification of neutrophil-derived exosomes as a new subcellular entity could result in elucidation of a molecular player providing a fundamental link between neutrophil-driven inflammation and multi-organ tissue damage observed in ENL with far-reaching implications for future research. Neutrophil-derived exosomes have already been demonstrated as a new means of intercellular communication promoting extracellular matrix destruction in an array of chronic inflammatory lung disease and have key immunomodulatory roles in a variety of inflammatory dermatological disorders, justifying their role as biomarkers indicating a variety of pathophysiological states (109–111).

Neutrophil-derived exosomes could be candidates for various clinical applications in leprosy. Analysis of exosomal content can reveal signature molecules including proteins and miRNAs, which might be relevant in detecting ENL susceptibility in LL patients, identifying patients who require prolonged treatment, and indicating the prognosis of immune reactions. In future endeavors, the functional and diagnostic potential of neutrophil-derived exosomal protein and/or miRNAs in leprosy could be investigated.

## Future Insights

- The utility of NLR in ENL needs to be tested in large prospective cohorts of multibacillary patients: the identification of adequate cut-off values or longitudinal evaluations over a prolonged treatment period is mandatory to determine its clinical usefulness.
- The phenotypical and functional divergence of neutrophil subpopulations in ENL needs further evaluation. Identifying subpopulations and related inflammatory mediators is of utmost significance within the context of applied immunology, as this allows for their application in clinical practice to assess disease activity, severity and the course of the reaction (acute vs. chronic).
- Investigations are required to elucidate the frequency and clinical significance of LDNs as well as expression of various activation surface markers to test their potential as a biomarker for ENL.
- The potential prognostic serum levels of NETs/NETs associated proteins should be evaluated in prospective follow up studies of ENL patients and its correlation with disease activity tested in larger cohorts. This could prove helpful in determination of the duration of therapy.
- How *M. leprae* interferes with metabolic pathways in neutrophils to precipitate ENL in LL patients merits further exploration.
- We support investigations into the concept of “*M leprae*-specific transcriptional-reprogramming” in neutrophils/NETs. Subsequent translation can yield altered molecular configurations that can be packaged into exosome vesicles and released out of NETs; such NET-associated exosomes may serve as potential “messengers/transmitters” that may cross talk with other active immune cells/tissue resident cells/Schwann cells resulting in the progressive fulminant multi-organ pathology seen in ENL.

## Open Questions

- What are the signals that cause neutrophil activation and subsequent development into LDNs following *M. leprae* infection?
- How do the newly recognized neutrophil subsets/subtypes/subpopulations (identified by phenotypic markers and segregated on basis of density centrifugation) contribute to ENL pathophysiology?
- Do these neutrophil subpopulations exhibit any differences in granule protein content and secretion in the context of ENL susceptibility?
- Does *M. leprae* alter metabolic configuration of neutrophils? How does it alter its metabolic configuration to precipitate immunological exacerbation in LL patients?
- How does *M. leprae* use its weaponry (lipid virulence factors) to exploit various host biological pathways to rewire the host innate immune/cytokine responses in ENL?

Many aspects related to ENL pathophysiology remain enigmatic. Answers to these questions have the potential to facilitate discovery of clinical useful biomarkers in the near future.

## Summary and Conclusion

Profound metabolic changes are posited to occur in neutrophils during normal physiological processes and under various pathophysiological conditions including *M. leprae* infection, more research is required to shed light on the signaling pathways governing these perturbations and to elucidate the intricate interactions among various metabolic programming pathways in neutrophils, with the hope that this basic research can translate into the development of biomarker discovery and novel therapeutics and diagnostic strategies. This might help to identify patients who are prone to develop recurrent or chronic/recalcitrant T2R and initiate them on to targeted therapy.

Neutrophils are the key players in ENL and have a potential role as biomarkers of disease outcome or as therapeutic targets. However, there is still much work to be done before they might be used as validated prognostic markers. Moreover, newer therapeutic agents targeting particularly neutrophil migration and mobilization without causing global immunosuppression could be designed.

**Figure 1:**
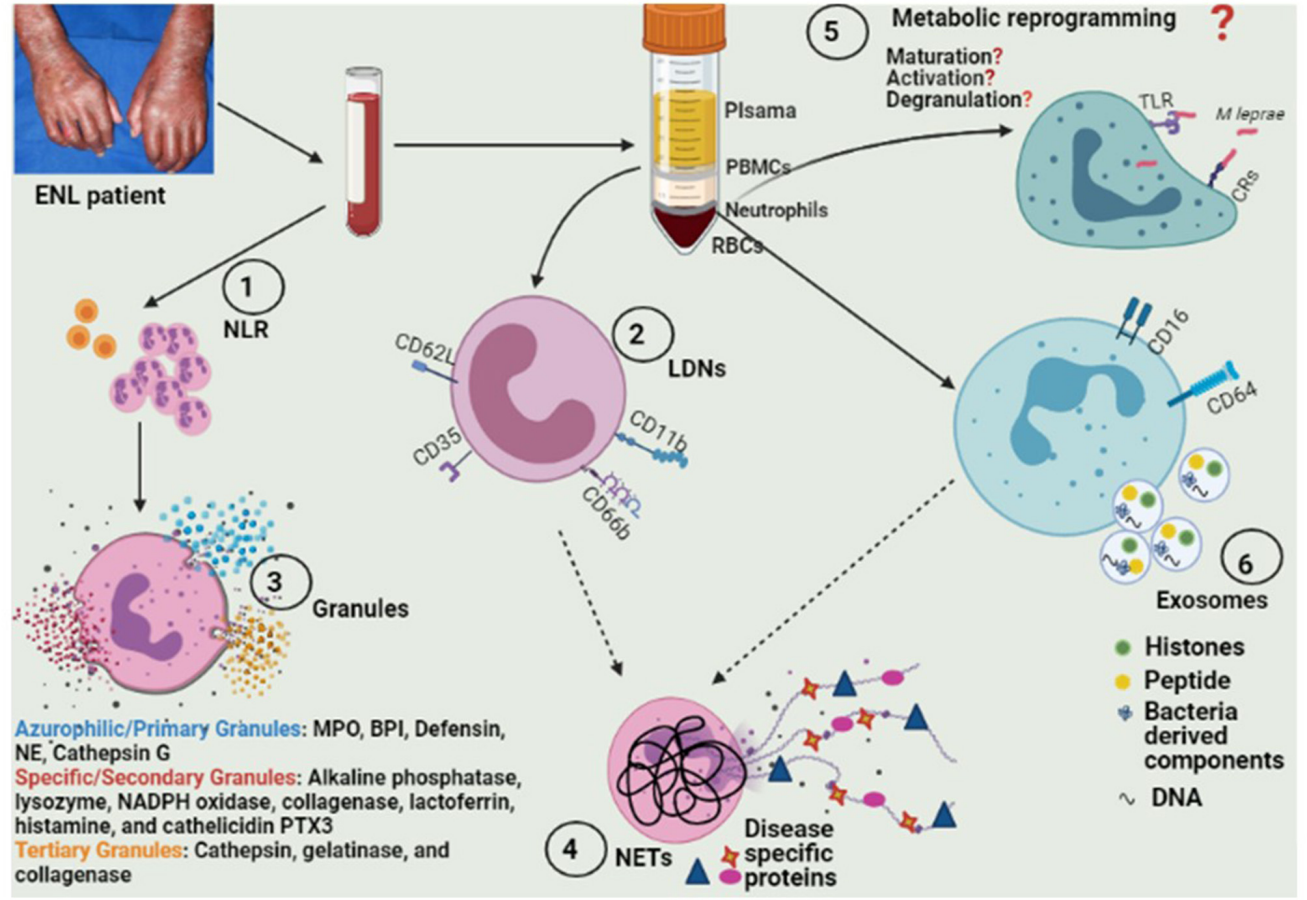
Model depicting various approaches for exploring different aspects of neutrophil biology for future biomarker discovery. (1) Identification of adequate cut-off values of NLR and their longitudinal evaluations over a prolonged treatment period in larger study cohorts. (2) Elucidation of frequency as well as the expression of various activation surface markers for LDNs (and determination of their clinical significance in ENL pathophysiology). (3) Assessment of differences in neutrophil granules' protein content and their secretion. (4) Evaluation of serum levels of NETs/NETs associated proteins in prospective follow up studies of ENL patients and their correlation with disease activity and also investigation of ENL-specific bioactive proteins loaded on NETs. (5) Exploration of the hypothesis of “*M leprae*-induced transcriptional-reprogramming” in neutrophils/NETs resulting in alterations in biosynthetic pathways associated with neutrophil maturation, activation, and degranulation. (6) Investigation of functional and diagnostic potential of neutrophil-derived exosomal protein and/or miRNAs. NLR, Neutrophil-lymphocyte ratio; LDNs, low density neutrophils; ENL, eryhtema nodosum leprosum; NETs, neutrophil extracelluar traps; TLRs, Toll like receptors; CRs, complement receptors; MPO, Myeloperoxidase; BPI, Bactericidal/permeability-increasing protein; NE, Neutrophil elastase; PTX3, Pentraxin-3.

Exploiting various aspects of neutrophil biology (Figure 1) can lead us to discover signature molecules that can be investigated as biomarkers for ENL. These biomarkers could also help us in diagnosis as well as prognostication of ENL patients.

We envision that liquid-biopsy based exosomal biomarkers for ENL can be developed and validated in the near future with the potential for enhanced early detection, improved diagnosis, disease outcome predictability, and therapeutic window determination.

Moreover, an integration of information from different “*-omics”* studies could provide scientists and clinicians with a powerful tool to understand the various aspects of neutrophil biology involved in development and progression of ENL. It will provide a great opportunity and hope to identify biomarkers specific for different cellular processes/states along with those indicating efficacious therapeutic intervention.

## Author Contributions

SS and KS: literature search, manuscript preparation, and experimental studies. MS: literature search and manuscript review. TN and SD: definition of intellectual content, manuscript editing, manuscript review, and clinical studies. RM: definition of intellectual content and manuscript review. SC: concepts, design, literature search, manuscript preparation, manuscript editing, and guarantor. All authors contributed to the article and approved the submitted version.

## Conflict of Interest

The authors declare that the research was conducted in the absence of any commercial or financial relationships that could be construed as a potential conflict of interest.

## References

[1] Lockwood DNJ. Chronic aspects of leprosy-neglected but important. Trans R Soc Trop Med Hyg. (2019) 113:812–16. 10.1093/trstmh/try131 30715525

[2] Walker SL Lockwood DNJ. Leprosy type 1 reactions and erythema nodosum leprosum. Artig Revisao. (2008) 83:75–82. 10.1590/S0365-05962008000100010

[3] Bhusan K Sunil D Inderjeet K. Epidemiological characteristics of leprosy reactions: 15 years experience from North India. Int J Lepr Other Mycobact Dis. (2004) 72:491. 10.1489/1544-581X(2004)072<0125:ECOLRY>2.0.CO;2 15301592

[4] Walker SL Lebas E Doni SN Lockwood DNJ Lambert SM. The mortality associated with erythema nodosum leprosum in Ethiopia: a retrospective hospital-based study. PLoS Negl Trop Dis. (2014) 8:e0002690. 10.1371/journal.pntd.0002690 24625394 PMC3953021

[5] Bhat RM Vaidya TP. What is new in the pathogenesis and management of erythema nodosum leprosum. Indian Dermatol Online J. (2020) 11:482–92. 10.4103/idoj.IDOJ_561_19 32832433 PMC7413435

[6] Walker SL Sales AM Butlin CR Shah M Maghanoy A Lambert SM. A leprosy clinical severity scale for erythema nodosum leprosum: an international, multicentre validation study of the ENLIST ENL severity scale. PLoS Negl Trop Dis. (2017) 11:e0005716. 10.1371/journal.pntd.0005716 28671966 PMC5510881

[7] Singal A. Current concepts and challenges in the management of erythema nodosum leprosum. Indian Dermatol Online J. (2020) 11:479–81. 10.4103/idoj.IDOJ_69_20 32832432 PMC7413424

[8] Kahawita IP Lockwood DNJ. Towards understanding the pathology of erythema nodosum leprosum. Trans R Soc Trop Med Hyg. (2008) 102:329–37. 10.1016/j.trstmh.2008.01.004 18313706

[9] Geluk A. Correlates of immune exacerbations in leprosy. Semin Immunol. (2018) 39:111–18. 10.1016/j.smim.2018.06.003 29950273

[10] Stefani MM Guerra JG Sousa ALM Costa MB Oliveira MLW Martelli CT. Potential plasma markers of type 1 and type 2 leprosy reactions: a preliminary report. BMC Infect Dis. (2009) 9:75. 10.1186/1471-2334-9-75 19473542 PMC2696458

[11] Gupta N Shankernarayan NP Dharmalingam K. α1-Acid glycoprotein as a putative biomarker for monitoring the development of the type II reactional stage of leprosy. J Med Microbiol. (2010) 59:400–7. 10.1099/jmm.0.016394-0 20075114

[12] Sengupta U. Alpha 1 acid glycoprotein: increased serum and localized mRNA expression as a monitor for reactions in leprosy. SOJ Microbiol Infect Dis. (2015) 3:1–4. 10.15226/sojmid/3/3/00137

[13] Schmitz V Berto R Garcia M Barbosa DM Pacheco S Machado ADM. Expression of CD64 on circulating neutrophils favoring systemic inflammatory status in erythema nodosum leprosum. PLoS Negl Trop Dis. (2016) 10:e0004955. 10.1371/journal.pntd.0004955 27556927 PMC4996526

[14] Mendes MA De Carvalho DS Amadeu TP Silva BJDA Prata RBDS Da Silva CO. Elevated pentraxin-3 concentrations in patients with leprosy: potential biomarker of erythema nodosum leprosum. J Infect Dis. (2017) 216:1635–43. 10.1093/infdis/jix267 29272525

[15] Hungria EM Bührer-Sékula S de Oliveira RM Aderaldo LC Pontes A de A Cruz R. Leprosy reactions: the predictive value of *Mycobacterium leprae*-specific serology evaluated in a Brazilian cohort of leprosy patients (U-MDT/CT-BR). PLoS Negl Trop Dis. (2017) 11:e0005396. 10.1371/journal.pntd.0005396 28222139 PMC5336302

[16] Negera E Walker SL Bekele Y Dockrell HM Lockwood N. Increased activated memory B-cells in the peripheral blood of patients with erythema nodosum leprosum reactions. PLoS Negl Trop Dis. (2017) 11:e0006121. 10.1371/journal.pntd.0006121 29253897 PMC5749895

[17] Negera E Walker SL Lema T Aseffa A Lockwood DN Dockrell HM. Complement C1q expression in Erythema nodosum leprosum. PLoS Negl Trop Dis. (2018) 12:e0006321. 10.1371/journal.pntd.0006321 29499046 PMC5851649

[18] Darmaputra IGN Herwanto N Rusyati LM Riawan W Endaryanto A Prakoeswa CRS. Distribution of iNOS expressions and TNF neutrofil cells as well as PGE2 and S100 Schwann cell dermal nerves in the erythema nodosum leprosum patients. Bali Med J. (2018) 7:262. 10.15562/bmj.v7i1.879

[19] Nobre L Nascimento LS Amorim FM Monteiro RG Freire-neto FP Carmo M. Differential immunoglobulin and complement levels in leprosy prior to development of reversal reaction and erythema nodosum leprosum. PLoS Negl Trop Dis. (2019) 13:e0007089. 10.1371/journal.pntd.0007089 30689631 PMC6366718

[20] Tavares IF dos Santos JB dos Santos Pacheco F Gandini M Meyer RM Moraes MO. Low density neutrophils as a potential biomarker of leprosy severity. J Immunol. (2020) 204:148–148. Available online at: http://www.jimmunol.org/content/204/1_Supplement/148.37.abstract (accessed May 1, 2020).

[21] Pacheco FS Berto R Brandão SS Ferreira H Rodrigues F Brandão J. Erythema nodosum leprosum neutrophil subset expressing IL-10R1 transmigrates into skin lesions and responds to IL-10. ImmunoHorizons. (2021) 4:47–56. 10.4049/immunohorizons.1900088 32034084

[22] Gomes LT Morato-Conceição YT Gambati AVM Maciel-Pereira CM Fontes CJF. Diagnostic value of neutrophil-to-lymphocyte ratio in patients with leprosy reactions. Heliyon. (2020) 6:e03369. 10.1016/j.heliyon.2020.e03369 32083213 PMC7021565

[23] Vilani-Moreno FR Brito-de-Souza VN Silva SMUR Barbosa ASAA Sartori BGC Campanelli AP. Increased serum levels of interleukin-6 in erythema nodosum leprosum suggest its use as a biomarker. Indian J Dermatol Venereol Leprol. (2021) 87:190. 10.25259/IJDVL_143_20 33769734

[24] McKenna E Mhaonaigh AU Wubben R Dwivedi A Hurley T Kelly LA. Neutrophils: need for standardized nomenclature. Front Immunol. (2021) 12:602963. 10.3389/fimmu.2021.602963 33936029 PMC8081893

[25] Rosales C. Neutrophil: a cell with many roles in inflammation or several cell types? Front Physiol. (2018) 9:1–17. 10.3389/fphys.2018.00113 29515456 PMC5826082

[26] Ley K Hoffman HM Kubes P Cassatella MA Zychlinsky A Hedrick CC. Neutrophils: new insights and open questions. Sci Immunol. (2018) 3:4579. 10.1126/sciimmunol.aat4579 30530726

[27] Wang WM Jin HZ. Role of neutrophils in psoriasis. J Immunol Res. (2020) 2020:18–23. 10.1155/2020/3709749 PMC7293746 32587871

[28] Mollinedo F. Neutrophil degranulation, plasticity, and cancer metastasis. Trends Immunol. (2019) 40:228–42. 10.1016/j.it.2019.01.006 30777721

[29] Schmitz V Tavares IF Pignataro P Machado ADM Pacheco S Schmitz V. Neutrophils in leprosy. Front Immunol. (2019) 10:495. 10.3389/fimmu.2019.00495 30949168 PMC6436181

[30] Drutz DJ Chen TSN Lu W-H. The continuous bacteremia of lepromatous leprosy. N Engl J Med. (1972) 287:159–64. 10.1056/NEJM197207272870402 4555967

[31] Chatterjee G Kaur S Sharma VK Vaishnavi C Ganguly NK. Bacillaemia in leprosy and effect of multidrug therapy. Lepr Rev. (1989) 60:197–−201. 10.5935/0305-7518.19890025 2682105

[32] Oliveira RB Moraes MO Oliveira EB Sarno EN Nery JAC Sampaio EP. Neutrophils isolated from leprosy patients release TNF-α and exhibit accelerated apoptosis *in vitro*. J Leukoc Biol. (1999) 65:364–71. 10.1002/jlb.65.3.364 10080541

[33] Goihman-Yahr M Rodriguez-Ochoa G Aranzazu N Pinardi ME de Gomez ME Ocanto A. *In vitro* activation of neutrophils by suspensions of *Mycobacterium leprae*. Int J Lepr. (1979) 47:570–4. 122626

[34] Goihman-yahr M Convit J Rodriguez-Ochoa G. NBT test in lepromatous leporosy. Lancet. (1973) 25:456–7. 10.1016/S0140-6736(73)92337-4 4124954

[35] Sher R Anderson R Glover A Wadee AA. Polymorphonuclear cell function in the various polar types of leprosy and erythema nodosum leprosum. Infect Immun. (1978) 21:959–65. 10.1128/iai.21.3.959-965.1978 711343 PMC422090

[36] Goihman-Yahr M Ulrich M Noya-León A Rojas A Convit J. Dermal exudate macrophages. Induction in dermal chambers and response to lymphokines. Clin Exp Immunol. (1975) 22:359–63. 1212821 PMC1538292

[37] Nauseef WM Borregaard N. Neutrophils at work. Nat Immunol. (2014) 15:2921. 10.1038/ni.2921 24940954

[38] Kamp VM Pillay J Lammers J-WJ Pickkers P Ulfman LH Koenderman L. Human suppressive neutrophils CD16 bright /CD62L dim exhibit decreased adhesion. J Leukoc Biol. (2012) 92:1011–20. 10.1189/jlb.0612273 22927481

[39] Pillay J Kamp VM Van Hoffen E Visser T Tak T Lammers JW. A subset of neutrophils in human systemic inflammation inhibits T cell responses through Mac-1. J Clin Invest. (2012) 122:327–36. 10.1172/JCI57990 22156198 PMC3248287

[40] Manz MG Boettcher S. Emergency granulopoiesis. Nat Rev Immunol. (2014) 14:302–14. 10.1038/nri3660 24751955

[41] Gomez D Farid S Malik HZ Young AL Toogood GJ Lodge JPA. Preoperative neutrophil-to-lymphocyte ratio as a prognostic predictor after curative resection for hepatocellular carcinoma. World J Surg. (2008) 32:1757–62. 10.1007/s00268-008-9552-6 18340479

[42] Azab B Bhatt VR Phookan J Murukutla S Kohn N Terjanian T. Usefulness of the neutrophil-to-lymphocyte ratio in predicting short- and long-term mortality in breast cancer patients. Ann Surg Oncol. (2012) 19:217–24. 10.1245/s10434-011-1814-0 21638095

[43] Cedrés S Torrejon D Martínez A Martinez P Navarro A Zamora E. Neutrophil to lymphocyte ratio (NLR) as an indicator of poor prognosis in stage IV non-small cell lung cancer. Clin Transl Oncol. (2012) 14:864–69. 10.1007/s12094-012-0872-5 22855161

[44] Torun S Tunc BD Suvak B Yildiz H Tas A Sayilir A. Assessment of neutrophil-lymphocyte ratio in ulcerative colitis: a promising marker in predicting disease severity. Clin Res Hepatol Gastroenterol. (2012) 36:491–97. 10.1016/j.clinre.2012.06.004 22841412

[45] Loonen AJM De Jager CPC Tosserams J Kusters R Hilbink M Wever PC. Biomarkers and molecular analysis to improve bloodstream infection diagnostics in an emergency care unit. PLoS ONE. (2014) 9:e0087315. 10.1371/journal.pone.0087315 24475269 PMC3903623

[46] Templeton AJ Mcnamara MG Šeruga B Vera-badillo FE Aneja P Ocaña A Leibowitz-amit R. Prognostic role of neutrophil-to-lymphocyte ratio in solid tumors : a systematic review and meta-analysis. J Natl Cancer Inst. (2014) 106:1–11. 10.1093/jnci/dju124 24875653

[47] Bhat T Bhat H Raza M Khoueiry G Meghani M Akhtar M. Neutrophil to lymphocyte ratio and cardiovascular diseases: a review. Expert Rev Cardiovasc Ther. (2013) 11:55–9. 10.1586/erc.12.159 23259445

[48] Mentis A-FA Kyprianou MA Xirogianni A Kesanopoulos K Tzanakaki G. Neutrophil-to-lymphocyte ratio in the differential diagnosis of acute bacterial meningitis. Eur J Clin Microbiol Infect Dis. (2016) 35:397–403. 10.1007/s10096-015-2552-1 26792137

[49] Kartal O At K. Value of neutrophil to lymphocyte and platelet to lymphocyte ratios in pneumonia. Bratislava Med J. (2017) 118:513–6. 10.4149/BLL_2017_099 29061056

[50] Djordjevic D Rondovic G Surbatovic M Stanojevic I Udovicic I Andjelic T. Neutrophil-to-lymphocyte ratio, monocyte-to-lymphocyte ratio, platelet-to-lymphocyte ratio, and mean platelet volume-to-platelet count ratio as biomarkers in critically ill and injured patients: which ratio to choose to predict outcome and nature of bacteremia? Mediators Inflamm. (2018) 2018:3758068. 10.1155/2018/3758068 30116146 PMC6079471

[51] Lee DJ Li H Ochoa MT Tanaka M Carbone RJ Damoiseaux R. Integrated pathways for neutrophil recruitment and inflammation in leprosy. J Infect Disesaes. (2010) 90095:558–69. 10.1086/650318 20070238 PMC2811231

[52] Leal-Calvo T Moraes MO. Reanalysis and integration of public microarray datasets reveals novel host genes modulated in leprosy. Mol Genet Genom. (2020) 295:1355–68. 10.1007/s00438-020-01705-6 32661593

[53] Inkeles MS Teles RMB Pouldar D Andrade PR Madigan CA Lopez D. Cell-type deconvolution with immune pathways identifies gene networks of host defense and immunopathology in leprosy. JCI Insight. (2016) 1:1–16. 10.1172/jci.insight.88843 27699251 PMC5033757

[54] Dupnik KM Bair TB Maia AO Amorim FM Costa MR Keesen TSL. Transcriptional changes that characterize the immune reactions of leprosy. J Infect Dis. (2015) 211:1658–76. 10.1093/infdis/jiu612 25398459 PMC4425823

[55] Mortaz E Alipoor SD Adcock IM Mumby S. Update on neutrophil function in severe inflammation. Front Immunol. (2018) 9:2171. 10.3389/fimmu.2018.02171 30356867 PMC6190891

[56] Hassani M Bongers S Hellebrekers P Hietbrink F Vrisekoop N Koenderman L. On the origin of low-density neutrophils. J Leukoc Biol. (2020) 107:809–18. 10.1002/JLB.5HR0120-459R 32170882 PMC7318192

[57] Xigris F. eCIRPing of low-density blood neutrophils in sepsis. J Leukoc Biol. (2020) 1:1–3. 10.1002/JLB.4CE0820-504R 33112444

[58] Silvestre-roig C Fridlender ZG Glogauer M Scapini P. Neutrophil diversity in health and disease. Trends Immunol. (2019) 40:565–83. 10.1016/j.it.2019.04.012 31160207 PMC7185435

[59] Carmona-rivera C Kaplan MJ. Low density granulocytes: a distinct class of neutrophils in systemic autoimmunity. Semin Immunopathol. (2013) 35:455–63. 10.1007/s00281-013-0375-7 23553215 PMC4007274

[60] van der Linden M van den Hoogen LL Westerlaken GHA Fritsch-Stork RDE van Roon JAG Radstake TRDJ. Neutrophil extracellular trap release is associated with antinuclear antibodies in systemic lupus erythematosus and anti-phospholipid syndrome. Rheumatol (United Kingdom). (2018) 57:1228–34. 10.1093/rheumatology/key067 29608758

[61] Denny MF Yalavarthi S Zhao W Thacker SG Anderson M Sandy AR. A distinct subset of proinflammatory neutrophils isolated from patients with systemic lupus erythematosus induces vascular damage and synthesizes type I IFNs. J Immunol. (2010) 184:3284–97. 10.4049/jimmunol.0902199 20164424 PMC2929645

[62] Bashant KR Aponte AM Randazzo D Rezvan Sangsari P Wood AJT Bibby JA. Proteomic, biomechanical and functional analyses define neutrophil heterogeneity in systemic lupus erythematosus. Ann Rheum Dis. (2021) 80:209–18. 10.1136/annrheumdis-2020-218338 32988843 PMC7855438

[63] Hidalgo A Chilvers ER Summers C Koenderman L. The neutrophil life cycle. Trends Immunol. (2018) 40:584–97. 10.1016/j.it.2019.04.013 31153737

[64] Rosales C. Neutrophils at the crossroads of innate and adaptive immunity. J Leukoc Biol. (2020) 108:377–96. 10.1002/JLB.4MIR0220-574RR 32202340

[65] Othman A Sekheri M Filep JG. Roles of neutrophil granule proteins in orchestrating inflammation and immunity. FEBS J. (2021). 10.1111/febs.15803. [Epub ahead of print]. 33683814 PMC9546106

[66] Teles RMB Teles RB Amadeu TP Moura DF Mendonça-Lima L Ferreira H. High matrix metalloproteinase production correlates with immune activation and leukocyte migration in leprosy reactional lesions. Infect Immun. (2010) 78:1012–21. 10.1128/IAI.00896-09 20008541 PMC2825932

[67] Youssef SS Attia EAS Awad NM Mohamed GF. Expression of matrix metalloproteinases (MMP-3 and MMP-9) in skin lesions of leprosy patients. J Egypt Women Dermatol Soc. (2009) 6:80–7. 10.118/1A1.00896-09

[68] Galkina SI Fedorova NV Golenkina EA Stadnichuk VI Sud'ina GF. Cytonemes versus neutrophil extracellular traps in the fight of neutrophils with microbes. Int J Mol Sci. (2020) 21:1–22. 10.3390/ijms21020586 31963289 PMC7014225

[69] Zhao X Yang L Chang N Hou L Zhou X Yang L. Neutrophils undergo switch of apoptosis to NETosis during murine fatty liver injury via S1P receptor 2 signaling. Cell Death Dis. (2020) 11:379. 10.1038/s41419-020-2582-1 32424179 PMC7235026

[70] Frangou E Chrysanthopoulou A Mitsios A Kambas K Arelaki S Angelidou I. REDD1/autophagy pathway promotes thromboinflammation and fibrosis in human systemic lupus erythematosus (SLE) through NETs decorated with tissue factor (TF) and interleukin-17A (IL-17A). Ann Rheum Dis. (2019) 78:238–48. 10.1136/annrheumdis-2018-213181 30563869 PMC6352428

[71] Mutua V Gershwin LJ. A review of neutrophil extracellular traps (NETs) in disease: potential anti-NETs therapeutics. Clin Rev Allergy Immunol. (2020) 1:1–8. 10.1007/s12016-020-08804-7 32740860 PMC7395212

[72] Sollberger G Tilley DO Zychlinsky A. Neutrophil extracellular traps: the Biology of chromatin externalization. Dev Cell. (2018) 44:542–53. 10.1016/j.devcel.2018.01.019 29533770

[73] Neubert E Meyer D Kruss S Erpenbeck L. The power from within - Understanding the driving forces of neutrophil extracellular trap formation. J Cell Sci. (2020) 133:241075. 10.1242/jcs.241075 32156720

[74] Daniel C Leppkes M Muñoz LE Schley G Schett G Herrmann M. Extracellular DNA traps in inflammation, injury and healing. Nat Rev Nephrol. (2019) 15:559–75. 10.1038/s41581-019-0163-2 31213698

[75] Tamas N Sperandio M Attila M. Neutrophils as emerging therapeutic targets. Nat Rev Drug Discov. (2020) 19:253–75. 10.1038/s41573-019-0054-z 31969717

[76] Fousert E Toes R Desai J. Neutrophil extracellular traps (NETs) take the central stage in driving autoimmune responses. Cells. (2020) 9:40915. 10.3390/cells9040915 32276504 PMC7226846

[77] Stakos D Skendros P Konstantinides S Ritis K. Traps N' Clots: NET-mediated thrombosis and related diseases. Thromb Haemost. (2020) 120:373–83. 10.1055/s-0039-3402731 31940675

[78] Da Silva CO Dias AA Da Costa Nery JA De Miranda MacHado A Ferreira H Rodrigues TF. Neutrophil extracellular traps contribute to the pathogenesis of leprosy type 2 reactions. PLoS Negl Trop Dis. (2019) 13:e0007368. 10.1371/journal.pntd.0007368 31504035 PMC6736252

[79] Yoshida GJ. Metabolic reprogramming: the emerging concept and associated therapeutic strategies. J Exp Clin Cancer Res. (2015) 34:1–10. 10.1186/s13046-015-0221-y 26445347 PMC4595070

[80] Kumar R Singh P Kolloli A Shi L Bushkin Y Tyagi S. Immunometabolism of phagocytes during mycobacterium tuberculosis infection. Front Mol Biosci. (2019) 6:1–20. 10.3389/fmolb.2019.00105 31681793 PMC6803600

[81] Shuvalov O Daks A Fedorova O Petukhov A Barlev N. Linking metabolic reprogramming, plasticity and tumor progression. Cancers (Basel). (2021) 13:1–25. 10.3390/cancers13040762 33673109 PMC7917602

[82] Xia L Oyang L Lin J Tan S Han Y Wu N. The cancer metabolic reprogramming and immune response. Mol Cancer. (2021) 20:1–21. 10.1186/s12943-021-01316-8 33546704 PMC7863491

[83] Guerra L Bonetti L Brenner D. Metabolic modulation of immunity: a new concept in cancer immunotherapy. Cell Rep. (2020) 32:107848. 10.1016/j.celrep.2020.107848 32640218

[84] Rice CM Davies LC Subleski JJ Maio N Gonzalez-Cotto M Andrews C. Tumour-elicited neutrophils engage mitochondrial metabolism to circumvent nutrient limitations and maintain immune suppression. Nat Commun. (2018) 9:5099. 10.1038/s41467-018-07505-2 30504842 PMC6269473

[85] Dai YD Dias P. Exosomes or microvesicles, a secreted subcellular organelle contributing to inflammation and diabetes. Diabetes. (2018) 67:2154–56. 10.2337/dbi18-0021 30348822

[86] Biadglegne F König B Rodloff AC Dorhoi A Sack U. Composition and clinical significance of exosomes in tuberculosis: a systematic literature review. J Clin Med. (2021) 10:145. 10.3390/jcm10010145 33406750 PMC7795701

[87] Spencer N Yeruva L. Role of bacterial infections in extracellular vesicles release and impact on immune response. Biomed J. (2021) 44:157–64. 10.1016/j.bj.2020.05.006 32888911 PMC8178569

[88] de Macedo CS Lara FA Pinheiro RO Schmitz V de Berrêdo-Pinho M Pereira GM. New insights into the pathogenesis of leprosy: contribution of subversion of host cell metabolism to bacterial persistence, disease progression, and transmission. F1000Research. (2020) 9:21383. 10.12688/f1000research.21383.1 32051758 PMC6996526

[89] Mattos KA Oliveira VCG Berrêdo-Pinho M Amaral JJ Antunes LCM Melo RCN. Mycobacterium leprae intracellular survival relies on cholesterol accumulation in infected macrophages: a potential target for new drugs for leprosy treatment. Cell Microbiol. (2014) 16:797–815. 10.1111/cmi.12279 24552180 PMC4262048

[90] Díaz Acosta CC Dias AA Rosa TLSA Batista-Silva LR Rosa PS Toledo-Pinto TG. PGL I expression in live bacteria allows activation of a CD206/PPARγ cross-talk that may contribute to successful Mycobacterium leprae colonization of peripheral nerves. PLOS Pathog. (2018) 14:e1007151. 10.1371/journal.ppat.1007151 29979790 PMC6056075

[91] De Macedo CS Anderson DM Pascarelli BM Spraggins JM Sarno EN Schey KL. MALDI imaging reveals lipid changes in the skin of leprosy patients before and after multidrug therapy (MDT). J Mass Spectrom. (2015) 50:1374–85. 10.1002/jms.3708 26634971

[92] Vallochi AL Teixeira L Oliveira K da S Maya-Monteiro CM Bozza PT. Lipid droplet, a key player in host-parasite interactions. Front Immunol. (2018) 9:1022. 10.3389/fimmu.2018.01022 29875768 PMC5974170

[93] Amaral JJ Antunes LCM de Macedo CS Mattos KA Han J Pan J. Metabonomics reveals drastic changes in anti-inflammatory/pro-resolving polyunsaturated fatty acids-derived lipid mediators in leprosy disease. PLoS Negl Trop Dis. (2013) 7:e2381. 10.1371/journal.pntd.0002381 23967366 PMC3744420

[94] Al-Mubarak R Vander Heiden J Broeckling CD Balagon M Brennan PJ Vissa VD. Serum metabolomics reveals higher levels of polyunsaturated fatty acids in lepromatous leprosy: potential markers for susceptibility and pathogenesis. PLoS Negl Trop Dis. (2011) 5:e0001303. 10.1371/journal.pntd.0001303 21909445 PMC3167790

[95] Medeiros RCA De Vasconcelos Girardi KDC Cardoso FKL De Siqueira Mietto B De Toledo Pinto TG Gomez LS. Subversion of schwann cell glucose metabolism by *Mycobacterium leprae*. J Biol Chem. (2016) 291:21375–87. 10.1074/jbc.M116.725283 27555322 PMC5076808

[96] Mattos KA Lara FA Oliveira VGC Rodrigues LS D'Avila H Melo RCN. Modulation of lipid droplets by *Mycobacterium leprae* in Schwann cells: a putative mechanism for host lipid acquisition and bacterial survival in phagosomes. Cell Microbiol. (2011) 13:259–73. 10.1111/j.1462-5822.2010.01533.x 20955239

[97] de Souza Sales J Lara FA Amadeu TP de Oliveira Fulco T da Costa Nery JA Sampaio EP. The role of indoleamine 2, 3-dioxygenase in lepromatous leprosy immunosuppression. Clin Exp Immunol. (2011) 165:251–63. 10.1111/j.1365-2249.2011.04412.x 21592112 PMC3142650

[98] Mellor AL Lemos H Huang L. Indoleamine 2,3-Dioxygenase and tolerance: where are we now? Front Immunol. (2017) 8:1360. 10.3389/fimmu.2017.01360 29163470 PMC5663846

[99] de Mattos Barbosa MG da Silva Prata RB Andrade PR Ferreira H de Andrade Silva BJ da Paixão de Oliveira JA. Indoleamine 2,3-dioxygenase and iron are required for Mycobacterium leprae survival. Microbes Infect. (2017) 19:505–14. 10.1016/j.micinf.2017.06.006 28684130

[100] Curi R Levada-Pires AC Silva EB Da Poma SDO Zambonatto RF Domenech P. The critical role of cell metabolism for essential neutrophil functions. Cell Physiol Biochem. (2020) 54:629–47. 10.33594/000000245 32589830

[101] Kumar S Dikshit M. Metabolic Insight of Neutrophils in health and disease. Front Immunol. (2019) 10:2099. 10.3389/fimmu.2019.02099 31616403 PMC6764236

[102] Wang M Yu F Ding H Wang Y Li P Wang K. Emerging function and clinical values of exosomal microRNAs in cancer. Mol Ther - Nucleic Acids. (2019) 16:791–804. 10.1016/j.omtn.2019.04.027 31163321 PMC6545365

[103] LeBleu VS Kalluri R. Exosomes as a multicomponent biomarker platform in cancer. Trends Cancer. (2020) 6:767–74. 10.1016/j.trecan.2020.03.007 32307267

[104] Goulielmaki E Ioannidou A Tsekrekou M Stratigi K Poutakidou IK Gkirtzimanaki K. Tissue-infiltrating macrophages mediate an exosome-based metabolic reprogramming upon DNA damage. Nat Commun. (2020) 11:42. 10.1038/s41467-019-13894-9 31896748 PMC6940362

[105] Jeannin P Chaze T Giai Gianetto Q Matondo M Gout O Gessain A. Proteomic analysis of plasma extracellular vesicles reveals mitochondrial stress upon HTLV-1 infection. Sci Rep. (2018) 8:1–7. 10.1038/s41598-018-23505-0 29581472 PMC5980083

[106] Fratini F Tamarozzi F Macchia G Bertuccini L Mariconti M Birago C. Proteomic analysis of plasma exosomes from cystic echinococcosis patients provides in vivo support for distinct immune response profiles in active vs inactive infection and suggests potential biomarkers. (2020) 14:e0008586. 10.1371/journal.pntd.0008586 33017416 PMC7535053

[107] Wang J Wang Y Tang L Garcia RC. Extracellular vesicles in mycobacterial infections: their potential as molecule transfer vectors. Front Immunol. (2019) 10:1929. 10.3389/fimmu.2019.01929 31474995 PMC6703136

[108] Ziegenbalg A Prados-Rosales R Jenny-Avital ER Kim RS Casadevall A Achkar JM. Immunogenicity of mycobacterial vesicles in humans: identification of a new tuberculosis antibody biomarker. Tuberculosis. (2013) 93:448–55. 10.1016/j.tube.2013.03.001 23562367 PMC3681920

[109] Vargas A Roux-Dalvai F Droit A Lavoie JP. Neutrophil-derived exosomes: A new mechanism contributing to airway smooth muscle remodeling. Am J Respir Cell Mol Biol. (2016) 55:450–61. 10.1165/rcmb.2016-0033OC 27105177

[110] Shao S Fang H Zhang J Jiang M Xue K Ma J. Neutrophil exosomes enhance the skin autoinflammation in generalized pustular psoriasis via activating keratinocytes. FASEB J. (2019) 33:1–16. 10.1096/fj.201802090RR 30811955

[111] Shao S Fang H Li Q Wang G. Extracellular vesicles in inflammatory skin disorders: from pathophysiology to treatment. Theranostics. (2020) 10:9937–55. 10.7150/thno.45488 32929326 PMC7481415

